# Platinum(II) Oxalato Complexes Involving Adenosine-Based *N*-Donor Ligands: Synthesis, Characterization and Cytotoxicity Evaluation

**DOI:** 10.3390/molecules19033832

**Published:** 2014-03-24

**Authors:** Pavel Štarha, Igor Popa, Zdeněk Trávníček

**Affiliations:** Regional Centre of Advanced Technologies and Materials, Department of Inorganic Chemistry, Faculty of Science, Palacký University, 17. listopadu 12, CZ-77146 Olomouc, Czech Republic; E-Mails: pavel.starha@upol.cz (P.Š.); igor.popa@upol.cz (I.P.)

**Keywords:** platinum(II) complexes, oxalato complexes, *N*6-benzyladenosine derivatives, multinuclear NMR, antitumor activity, DFT calculations

## Abstract

A one-step synthetic procedure using the reaction of potassium bis(oxalato)platinate(II) with the corresponding *N*6-benzyladenosine derivative (*n*L) provided the [Pt(ox)(*n*L)_2_]∙1.5H_2_O oxalato (ox) complexes **1**–**5**, involving the *n*L molecules as monodentate coordinated *N*-donor ligands. The complexes were thoroughly characterized by elemental analysis, multinuclear (^1^H, ^13^C, ^15^N, ^195^Pt) and two dimensional NMR, infrared and Raman spectroscopy, and mass spectrometry, proving their composition and purity as well as coordination of *n*L through the N7 atom of the purine moiety. Geometry of [Pt(ox)(*4F*L)_2_] (**5**) was optimized at the B3LYP/LANLTZ/6-311G** level of theory. The complexes were screened for their *in vitro* cytotoxicity against two human cancer cell lines (HOS osteosarcoma and MCF7 breast adenocarcinoma), but they did not show any effect up to the concentration of 50.0 µM (compounds **1**, **2**) or 20.0 µM (compounds **3**–**5**).

## 1. Introduction

Adenosine is considered to be a biologically important molecule because it is involved in the structure of nucleic acids or adenosine-phosphates (ATP, ADP, AMP) necessary for the intracellular transfer and storage of energy. From the platinum-based anticancer therapy point of view, adenosine represents one of the targets within the DNA molecule (the formation of 1,2-intrastrand adenine-guanine adducts) [1], which has been intensively studied on adenosine or adenosine-involving oligonucleotides, both used as model systems for the studies of the mechanism of action of the platinum-based antitumor active substances [2,3]. Surprisingly, to the best of our knowledge, only two papers dealing with platinum(II) complexes involving adenosine-based *N*-donor ligands have been published to date. Cleare and Hoeschele reported the *in vivo* cytotoxicity of a platinum(II) dichlorido complex with the adenosine ligand against mice sarcoma 180 cells [4], and besides that we reported a series of *trans*-platinum(II) dichlorido complexes involving *N*6-benzyladenosine-based ligands [5].

Our group has been interested in the study of antitumor active transition metal complexes with *N*6-benzyladenine-based *N*-donor ligands for many years (see e.g., [5,6,7,8] and the references cited therein). In general, some *N*6-benzyladenine derivatives represent an interesting group of compounds showing biological, and in particular cytotoxic activity. A representative of this group of compounds, 2-(1-ethyl-2-hydroxyethylamino)-*N*6-benzyl-9-isopropyladenine (roscovitine), is currently in the IIb phase of the clinical trials against non-small cell lung cancer [9,10]. On the other hand, the 2-chloro-*N*6-benzyl-9-isopropyladenine derivatives (synthetic precursors of roscovitine and its substituted-benzyl derivatives) were found to be cytotoxically inactive. Interestingly, the most *in vitro* cytotoxic platinum(II) complexes involving *N*6-benzyladenine derivatives were not those involving the highly antitumor effective *N*6-benzyladenine derivatives, but the complexes bearing the mentioned biologically inactive 2-chloro-*N*6-benzyl-9-isopropyladenine derivatives [11]. In other words, it is difficult (if possible) to find rational structure-activity relationships between the composition of the final platinum(II) complexes and biological profile of the *N*6-benzyladenine derivatives themselves. It has to be noted that it was just the platinum(II) oxalato complexes involving the mentioned inactive 2-chloro-*N*6-benzyl-9-isopropyladenine derivatives, which showed the highest cytotoxic activity [11] and which exceeded the cytotoxicity of the other types (*i.e.*, dichlorido [12] or cyclobutan-1,1'-dicarboxylato [13]) of the studied platinum(II) complexes.

The objective of this work was to study whether the formation of platinum(II) oxalato complexes involving the *n*L derivatives as *N*-donor carrier ligands {*note*: we chose four *n*L derivatives (*2OMe*L, *2Cl*L, *4OMe*L and *4Me*L) reported as inactive (IC_50_ > 166.7 µM) on HOS and MCF7, and one compound (*4F*L) effective against both the mentioned cell lines (IC_50_ = 13.2 µM against HOS and 21.0 µM against MCF7) [14]} provides more or less *in vitro* cytotoxic species as compared to the starting *n*L derivatives and to the previously published *trans*-platinum(II) dichlorido complexes with the same *N*-donor ligands [5].

## 2. Results and Discussion

**Figure 1:**
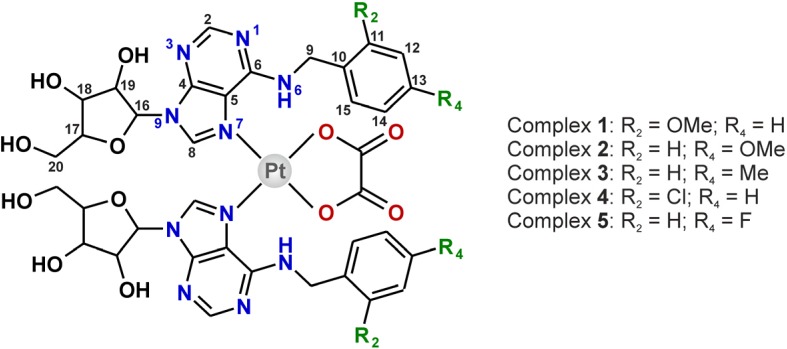
Structural formula of the prepared platinum(II) oxalato complexes [Pt(ox)(*n*L)_2_]·1.5H_2_O **1**–**5** given together with the atom numbering scheme.

Although the use of bis(oxalato)platinate(II) as the starting salt allow us to perform the syntheses at higher temperature (60 °C; further heating led to the [Pt(ox)_2_]^2−^ decomposition after several hours), the preparation of the title compounds was quite time-consuming (7 days in total). The products (yields >80%) were simply collected by filtration and washed by distilled water and methanol with no further purification needed (as proved by ^1^H-NMR). The empirical formulas [Pt(ox)(*n*L)_2_]·1.5H_2_O (**1**–**5**; Figure 1), followed from the results of elemental, thermogravimetric (TG) and differential thermal (DTA) analyses (see Figure S1 in Supplementary Materials). Simultaneous TG/DTA analyses revealed the presence of uncoordinated water molecules in **1**–**5**, which is evident from weight losses observed on the TG curves and *endo*-effects detected on DTA curves, with minima at *ca* 60 °C.

### 2.1. IR and Raman Spectroscopy

Both types of ligands (*i.e.*, *N*6-benzyladenosine derivatives and oxalate dianion) involved in the structures of **1**–**5** were detected by IR and Raman spectroscopy (see Figure S2 in Supplementary Materials). In connection with the fact that IR and Raman peaks characteristic for the corresponding vibrations were found within the same regions, the below given data represent the results obtained by both methods (for concrete results see Supplementary Materials). The presence of the oxalate dianion is proved by the maxima of ν_as_(C=O) at 1,669–1,714 cm^−1^ and ν_s_(C–O) at 1,366–1,371 cm^−1^ [15]. The coordination of the oxalate dianion to the central Pt(II) atom is connected with the peaks at 454–458 cm^−1^, assignable to the PtO_2_C_2_ ring deformation vibrations, and at 558–572 cm^−1^, which can be attributed to the stretching Pt–O vibrations. Similarly, the maxima of *ν*(Pt–N) observed at 505–520 cm^−1^ indicate the coordination of the *n*L molecules through nitrogen atoms. Other peaks connected with the presence of *n*L in **1**–**5** were detected in the 660–900 cm^−1^ and 1,450–1,590 cm^−1^ ranges (the skeletal vibrations of the purine moiety), between 1,049 and 1,081 cm^−1^ (*ν*(C–O)_aliphatic_ of the ribose moiety), at 1,610–1,614 cm^−1^ (*ν*(C–N)_aromatic_ vibrations) and at 3,116–3,129 cm^−1^ (*ν*(C–H)_aromatic_ vibration) [16,17]. The *ν*(N–H) vibration of the *n*L ligands, *ν*(O–H) vibration of the ribose moiety and/or *ν*(O–H) vibration of the water molecules of crystallization, which are also present in the structures of **1**–**5**, are overlapped to one broad peak with maxima between 3,304 and 3,326 cm^−1^. The peaks assignable to the phenyl ring substitution were detected at 1,239 cm^−1^ (*ν*(C–O)_aromatic_ for **1** and **2**), 1,182 cm^−1^ (*ν*(C–Cl)_aromatic_ for **4**) and 1,217 cm^−1^ (*ν*(C–F)_aromatic_ for **5**) [18].

### 2.2. ESI Mass Spectrometry

**Figure 2:**
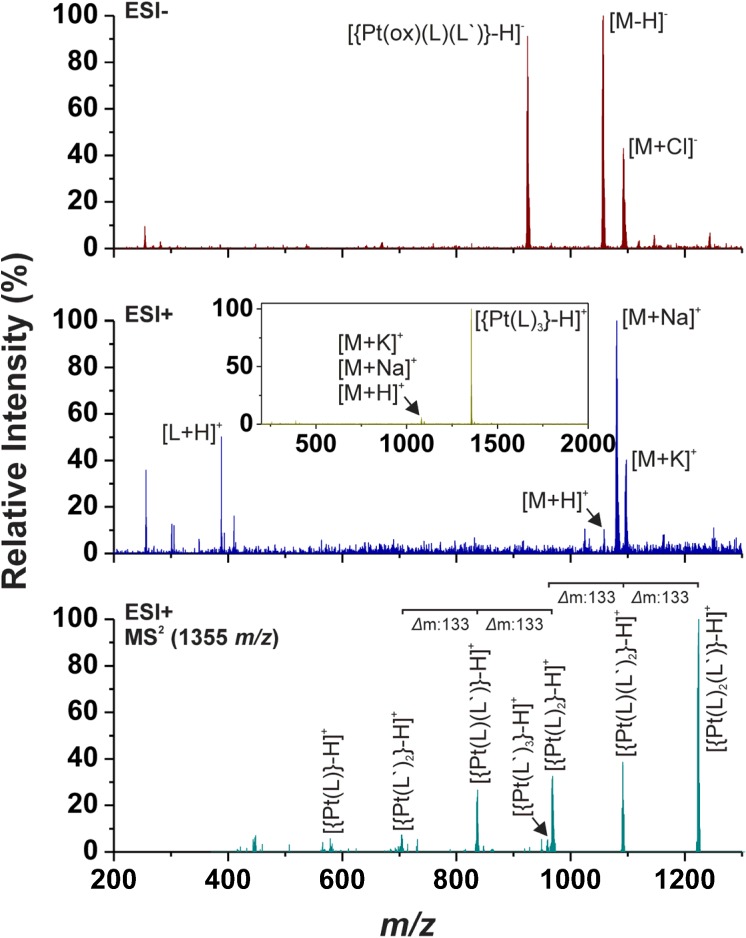
The results of mass spectrometry of **1** as obtained in the negative (ESI-; *up*) and positive (ESI+; *middle*; *inset*: spectrum obtained in the 200–2,000 *m/z* range) mode within the 200–1,300 *m/z* range. The MS^2^ spectrum of the [{Pt(L)_3_}–H]^+^ peak observed at 1,355 *m/z* showing the fragmentation of the species through the ribose moiety elimination (*down*).

Although the [{Pt(ox)(*n*L)_2_}+H]^+^ molecular peak was detected only in the ESI+ mass spectra of **1**, the adducts whose masses correspond to [{Pt(ox)(*n*L)_2_}+Na]^+^ and [{Pt(ox)(*n*L)_2_}+K]^+^ were found in the spectra of all the studied complexes measured in the 200–1,300 *m/z* range (Figure 2). The ESI+ spectra also contain the peaks of the [*n*L+H]^+^ fragments. Negative-mode electrospray mass spectrometry (ESI-) detected [{Pt(ox)(*n*L)_2_}–H]^−^ molecular peaks of **1**–**5**, and the peaks of deprotonated *n*L ligands, [*n*L–H]^−^, were also observed in the case of **3**–**5** (Figure 2). Next one intensive peak was found in the ESI- MS spectra of the studied complexes, whose mass corresponds to [{Pt(ox)(*n*L)(*n*L')}–H]^−^ (Figure 2), where *n*L' symbolizes the appropriate adenine derivative without ribose [19].

The above-mentioned fragmentation of the studied complexes through the cleavage of the ribose substituent was also observed in the positive-ion spectra [19] (Figure 2). Concretely, the ESI+ spectra measured up to 2,000 *m/z* contain the intensive peaks belonging to the [{Pt(*n*L)_3_}–H]^+^ species with several times higher intensity as compared with the above-discussed [{Pt(ox)(*n*L)_2_}+H]^+^, [{Pt(ox)(*n*L)_2_}+Na]^+^ and [{Pt(ox)(*n*L)_2_}+K]^+^ ones. The MS^2^ spectra of this species contained the series of peaks, which should be associated with the formation of the following species: [{Pt(*n*L)_3_]–H]}^+^, [{Pt(*n*L)_2_(*n*L')}–H]^+^, [{Pt(*n*L)(*n*L')_2_}–H]^+^, [{Pt(*n*L)_2_}–H]^+^, [{Pt(*n*L')_3_}–H]^+^, [{Pt(*n*L)(*n*L')}–H]^+^, [{Pt(*n*L')_2_}–H]^+^ and [{Pt(*n*L)}–H]^+^.

### 2.3. Multinuclear NMR Spectroscopy

**Figure 3:**
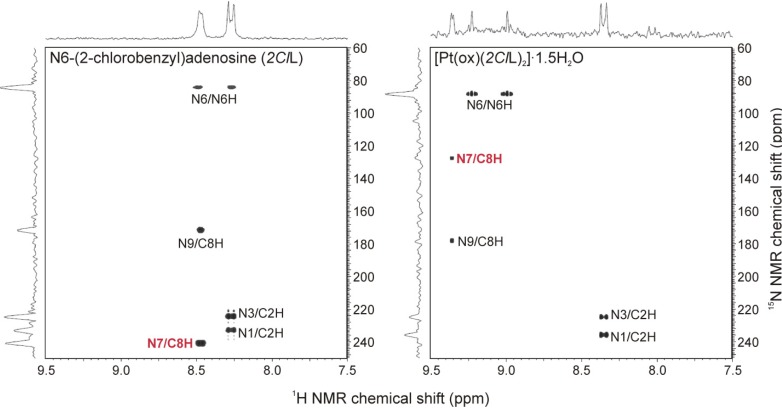
The results of ^1^H–^15^N gs-HMBC experiments on *N*6-(2-chlorobenzyl)adenosine (*2Cl*L; *left*) and the complex **4** (*right*) involving *2Cl*L as N7-coordinated ligand, showing the ^15^N NMR chemical shift changes caused by the coordination of the organic derivative to the Pt(II) atom and proving the mentioned N7-coordination mode of the adenosine-based ligands.

All the signals belonging to hydrogen, carbon and nitrogen atoms of both types of ligands involved in the structures of the studied complexes were found in appropriate NMR spectra and they were interpreted by means of ^1^H–^1^H gs-COSY, ^1^H–^13^C gs-HMQC, ^1^H–^13^C gs-HMBC and ^1^H–^15^N gs-HMBC experiments (see Figures S3 and S4 in Supplementary Materials). The ^1^H, ^13^C and ^15^N signals of free *n*L molecules were shifted in the appropriate spectra of **1**–**5** due to their coordination to the Pt(II) atom (see Synthesis section). Focusing on the adenine moiety, we summarized the coordination shifts (calculated as *Δδ* = *δ_complex_* − *δ_ligand_*) in Table 1. The obtained results unambiguously proved the coordination of the *n*L ligands through the N7 atom of the adenine moiety. Concretely, the ^15^N-NMR |*Δδ*| values calculated for the N7 atom equalled *ca* 113 ppm, while those of the other nitrogen atoms did not exceed 7 ppm (Figure 3; Table 1). The results of ^13^C-NMR spectroscopy were in compliance with the ^15^N-NMR spectroscopy, because the electron density redistribution caused by the coordination of the N7 atom to the Pt(II) atom led to the considerable shifts (|*Δδ*| > 3 ppm) of the C5 and C8 atoms adjacent to the mentioned N7 atom.

**Table 1:** The ^1^H, ^13^C and ^15^N NMR coordination shifts (*Δδ* = *δ_complex_* − *δ_ligand_*; ppm) of the adenine moiety atoms of the prepared complexes.

	^1^H-NMR				^13^C-NMR				^15^N NMR				
	C2H	N6H	C8H	C2H	C4	C5	C6	C8	N1	N3	N6	N7	N9
**1**	0.11	0.84	0.82	0.40	−0.87	−3.75	−1.57	3.14	3.10	−0.01	4.23	−113.28	6.68
**2**	0.06	0.63	0.78	0.15	−0.88	−3.77	−1.38	3.12	4.17	0.52	3.5	−113.11	6.40
**3**	0.07	0.67	0.81	0.32	−0.75	−3.65	−1.35	3.22	4.12	0.38	2.61	−113.06	6.73
**4**	0.08	0.73	0.89	1.31	−0.85	−3.63	−2.38	3.00	2.85	−0.16	2.87	−113.47	5.77
**5**	0.08	0.67	0.81	1.47	−0.88	−3.77	−2.67	3.14	3.33	−0.75	3.17	−113.46	6.46

In the case of ^1^H-NMR, the C8–H signal of **1**–**5** was strongly shifted downfield. However, the *Δδ* values of N6–H were found to be comparable or, in the case of **1**, even higher as compared with those of the mentioned C8–H protons. This is most likely the consequence of the mutual steric hindrance of both the coordinated *n*L molecules combined with intramolecular interaction of the N6–H proton.

The single signal, proving the symmetric bidentate coordination of the oxalate dianion to the Pt(II) atom, was detected at 165.53–165.81 ppm in the ^13^C-NMR spectra of the studied complexes. The ^195^Pt chemical shifts equal *ca* −1,690 ppm (for the detailed information see the section *Synthesis*).

### 2.4. Quantum Chemical Calculations

**Figure 4:**
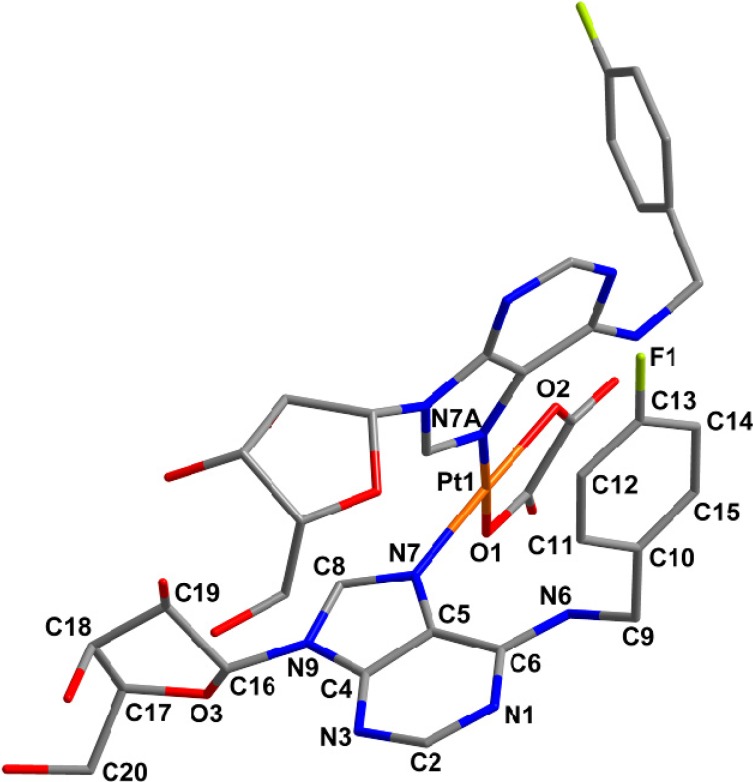
Geometry of [Pt(ox)(*4F*L)_2_] (**5**) optimized using dispersion corrected DFT-D3 quantum chemical calculations performed at the B3LYP/LANLTZ/6-311G** level of theory. Hydrogen atoms are omitted for clarity.

Several attempts to prepare single crystals of **1**–**5** suitable for a single crystal X-ray analysis were performed, but all of them were unsuccessful. That is why the structural aspects of the studied complexes, particularly the complex **5** as a representative one (Figure 4), were performed by means of DFT and dispersion corrected DFT-D3 [20,21] quantum chemical calculations performed at the B3LYP/LANLTZ/6-311G** [22,23,24,25] level of theory using the ORCA software package [26].

The structure of [Pt(ox)(*4F*L)_2_] (**5**) contains tetracoordinated central Pt(II) atom with two monodentate adenosine-based ligands coordinated through their N7 atoms and one bidentate oxalate dianion (see Table 2 for bond lengths), thus adopting the PtN_2_O_2_ donor set. A comparison of the calculated Pt–N and Pt–O bond lengths (Table 2) with those of four platinum(II) oxalato complexes involving two monodentate *N*-donor ligands (ammonia [27], 7-azaindole [28], 2-chloro-*N*6-(2,4-dimethoxybenzyl)-9-isopropyladenine (L_1_) [29] and 1-aminoethanol [30] deposited in the Cambridge Structural Database (CSD ver. 5.34, May 2013 update [31]), whose average values were found to be 2.016 Å (for Pt–O; 1.994–2.033 Å range) and 2.015 Å (for Pt–N; 2.001–2.050 Å range) indicated that the mentioned parameters correlate well in the case of the Pt–O bonds, but the Pt–N bond lengths calculated for **5** differ from those crystallographically determined. The N7–Pt–N7A bond angle formed by both the *4F*L molecules coordinated to the metal centre (Table 2) is again higher as compared with the mentioned platinum(II) oxalato complexes deposited in CSD (average of 91.05°). On the other hand its value does not differ considerably from that of the only crystallographically characterized platinum(II) complex with two adenosine-based ligands, *i.e.*, *cis*-[Pt(NH_3_)_2_(ado)_2_](ClO_4_)_2_∙3.5H_2_O (see Table 2); ado = adenosine [32]. This shows on the influence of the bulky ligand (ado or *4F*L in the case of this work) on the N7–Pt–N7A bond angle value or, in other words, on the degree of square-planar geometry deformation. A dihedral angle formed by the purine rings is another relevant parameter describing the molecular structure of the studied complex. We compared the value of 70.6° calculated for [Pt(ox)(*4F*L)] (**5**) with the mentioned *cis*-[Pt(NH_3_)_2_(ado)_2_]^2+^ complex (82.9(3)°) and with the platinum(II) oxalato complex [Pt(ox)(L_1_)]∙2DMF with differently substituted adenine-based ligands (83.1(6)°).

**Table 2:** Selected interatomic parameters as DFT and DFT-D3 calculated (B3LYP functional, LANLTZ/6-311G** basis sets) for the complex [Pt(ox)(*4F*L)] (**5**) and their comparison with those crystallographically determined for *cis*-[Pt(NH_3_)_2_(ado)_2_](ClO_4_)_2_∙3.5H_2_O(ado = adenosine; ref. [32]) and [Pt(ox)(L_1_)]∙2DMF (L_1_ = 2-chloro-*N*6-(2,4-dimethoxybenzyl)-9-isopropyladenine; ref. [29]).

Parameter	[Pt(ox)(*4F*L)] (5) ^a^	[Pt(ox)(*4F*L)] (5) ^b^	[Pt(NH_3_)_2_(ado)_2_]^2+^	[Pt(ox)(L_1_)]
Pt–N7	2.054	2.053	2.046(11)	2.001(3)
Pt–N7A	2.103	2.105	2.022(11)	2.001(3)
Pt–O1	2.011	2.012	–	1.994(2)
Pt–O2	2.019	2.017	–	2.010 (2)
N7–Pt–N7A	91.91	90.66	91.90(4)	89.74(11)
O1–Pt–O2	82.46	82.40	–	83.90(9)
N7–Pt–O2	172.41	173.19	–	175.93 (10)
N7A–Pt–O1	176.19	176.54	–	176.98 (10)

^a^ optimized at B3LYP/LANLTZ/6-311G**; ^b^ optimized at B3LYP/LANLTZ/6-311G** with DFT-D3 dispersion correction.

A significant difference is the most probably connected, besides the chemical differences of the bulky *N*-donor ligands, with the fact that the DFT calculations were performed in vacuum and thus, intermolecular non-covalent contacts were ignored. Therefore, we also performed the calculation using the DFT-D3 dispersion correction and found, as anticipated, that bond lengths were influenced insignificantly only, however, on the other side angles in the vicinity of the platinum(II) atom as well as mutual orientations of purine and PtN_2_O_2_ moieties are influenced fundamentally, as can be demonstrated by dihedral angles between the following least-squares planes: purine 1 (involving the N7 atom) *vs.* purine 2 (involving the N7A atom): 70.56° (DFT), 75.40° (DFT-D3); purine 1 *vs.* PtN_2_O_2_: 25.19° (DFT), 19.09° (DFT-D3); and purine 2 *vs.* PtN_2_O_2_: 68.88° (DFT), 67.95° (DFT-D3).

### 2.5. In Vitro Cytotoxicity

The free *N*6-benzyladenosine derivatives (*n*L), used in this work as *N*-donor ligands of platinum(II) oxalato complexes, were formerly studied [14] by an acetoxymethyl (AM) assay up to the concentration of 166.7 µM on the HOS and MCF7 (and several others) cells, as described above in the Introduction. Herein it has to be noted, that although it is known that the type of assay used should not affect the obtained activity results [33], we tested the *n*L substances once again using an MTT assay (which we use in our laboratory for *in vitro* cytotoxicity testing) to make the results obtained for *n*L comparable with those of **1**–**5** tested by the same (MTT) assay. Moreover, we studied the *in vitro* cytotoxicity of *n*L and **1**–**5** only to the concentration of 50.0 µM (not to 166.7 µM), because, as generally accepted, testing to the higher concentration limit is not meaningful from the general biological/therapeutic point of view. Surprisingly, the obtained results showed all the *n*L derivatives as being inactive within the studied concentration range (*i.e.*, with IC_50_ > 50.0 µM), which is in contradiction with the moderate *in vitro* cytotoxicity against HOS (IC_50_ = 13.2 µM) and MCF7 (IC_50_ = 21.0 µM) reported for *4F*L in the literature [14]. The correctness of the results obtained on *n*L compounds by an MTT assay, as reported in this work, could be proved by a comparison with the results determined by the same method on the MCF7 cells for the standard cisplatin (IC_50_ = 18.1 ± 5.1 µM, see below), which correspond well with pIC_50_ = 4.75 (*i.e.*, IC_50_ = *ca* 17.8 µM) reported by Mueller *et al.* [33].

The prepared complexes **1**–**5** were tested for their *in vitro* cytotoxicity against HOS osteosarcoma and MCF7 breast adenocarcinoma human cancer cell lines. The complexes were found to be differently soluble in the used medium (a water/DMF mixture, with a maximal DMF concentration of 0.1%). Concretely, the complexes **1** and **2** were soluble up to the tested concentration limit (50.0 µM), while **3**–**5** showed lower solubility and precipitated from the solutions with the concentrations higher than 20.0 µM. Regardless of solubility of the studied complexes, we did not observe any biological effect against the tumour cells (IC_50_ > 50.0 µM for **1** and **2**, and >20.0 µM for **3**–**5**). The reference standard, cisplatin, showed moderate *in vitro* cytotoxicity with IC_50_ = 25.4 ± 8.5 µM against HOS and 18.1 ± 5.1 µM against MCF7.

Herein we have to mentioned that one of the objectives of this work was to study whether the cytotoxicity of the starting adenosine derivatives *n*L themselves may affects the cytotoxic activity of the resulting platinum(II) oxalato complexes involving these substances as *N*-donor carrier ligands. We were also interested in comparison of **1**–**5**, with *N*-donor ligands mutually arranged in *cis*-position, with the recently described *trans*-dichloridoplatinum(II) complexes with the same *N*-donor ligands, which were found as antitumor inactive up to 50.0 µM on HOS and MCF7 cancer cell lines (*i.e.*, IC_50_ > 50.0 µM) [5]. Since no cytotoxicity was found on both the free adenosine derivatives (*n*L) and the complexes **1**–**5**, no comparison regarding the effect of platination on the studied biological properties of the *N6*-benzyladenosine derivatives can be made. In other words, it may be concluded that the formation of the platinum(II) oxalato complexes **1**–**5** involving cytotoxically inactive *n*L derivatives does not lead to cytotoxic species. Besides that, we may also conclude that the variations of biologically relevant leaving groups (*i.e.*, chloride *vs.* oxalate) as well as changes in geometry (*i.e.*, *trans vs. cis*) of the platinum(II) complexes involving *n*L have no impact on the cytotoxicity against HOS and MCF7 human cancer cell lines.

### 2.6. Cellular Accumulation Study

With respect to the generally known fact, that the *in vitro* cytotoxic action of platinum complexes depends on the cellular accumulation rate [34], we evaluated cellular uptake of the representative complex **5** and cisplatin by MCF7 human cancer cells (due to their known sensitivity to cisplatin and platinum complexes in general), in order to explain lack of *in vitro* cytotoxic effect of **1**–**5**. The obtained ICP-MS results clearly indicated about 50-fold lower cellular uptake of the studied complex **5** (0.9 ng/10^6^ cells) as compared with cisplatin (47 ng/10^6^ cells), which correlates with the above-discussed *in vitro* cytotoxicity results showing **5** as inactive contrary to cisplatin having IC_50_ on MCF7 cells equal to 18.1 µM.

### 2.7. ESI-MS and NMR Studies on Hydrolysis

Since the hydrolysis of the antitumor active platinum(II) complexes, a crucial step within the mechanism of action [35], is usually based on replacement of leaving groups (*i.e.*, the oxalato ligands in the case of **1**–**5**) leading to activated and more reactive aqua- and/or hydroxidoplatinum(II) species, we studied the ability of the representative complex **5** to undergo hydrolysis in water-containing solution (*i.e.*, a water/methanol mixture). Although we detected several new peaks in the mass spectra of the water/methanol mixture solution of **5** in comparison with the spectra of **5** dissolved in pure methanol, their isotopic distribution did not correspond to that of platinum-containing species (Figure S5). In other words, no new platinum-containing species was found in the mass spectra recorded on the water/methanol solution of **5** even after 48 h, which excluded the hydrolysis of **5**.

^1^H and ^195^Pt NMR spectroscopy were used to investigate the behaviour of the representative complex **5** in DMF-*d_7_*/H_2_O mixture (4:1 *v/v*). The hydrolysis of the platinum(II) oxalato complexes, known to be necessary for their cytotoxic action, should be connected with opening of the PtO_2_C_2_ ring and/or substitution of the oxalate dianion by two water molecules or OH^−^ ions, both resulting in the change of inner coordination sphere and electron density within the initial complex, which is known to provide different ^1^H- and ^195^Pt-NMR chemical shifts [36]. Since we did not observe any new signals in both the ^1^H- and ^195^Pt-NMR spectra (Figure S6), it can be concluded, analogically to the above-mentioned ESI-MS experiments, that **5** does not undergo any changes within the structure during 48 h in the water-containing solution.

## 3. Experimental

### 3.1. Materials and Methods

K_2_[PtCl_4_], K_2_(ox)·H_2_O and solvents were purchased from Sigma‑Aldrich Co. (Prague, Czech Republic) and Acros Organics Co. (Pardubice, Czech Republic) and they were used as received. K_2_[Pt(ox)_2_]∙2H_2_O was prepared according to the literature [29], while the *N*6-benzyladenosine derivatives (*n*L), namely *N*6-(2-methoxybenzyl)adenosine (*2OMe*L), *N*6-(4-methoxybenzyl)adenosine (*4OMe*L), *N*6-(4-methylbenzyl)adenosine (*4Me*L), *N*6-(2-chlorobenzyl)adenosine (*2Cl*L), *N*6-(4-fluorobenzyl)adenosine (*4F*L), were obtained as described in ref. [37].

Elemental analysis was performed on a Flash 2000 CHNS Elemental Analyzer (Thermo Scientific, Waltham, MA, USA). IR spectra were recorded by an ATR technique on a Nexus 670 FT-IR (Thermo Nicolet) in the 150–600 cm^−1^ and 400–4000 cm^−1^ region. Raman spectra were measured by an NXR FT-Raman Module (Thermo Nicolet, Waltham, MA, USA) at 150–3750 cm^−1^. Simultaneous thermogravimetry and differential thermal analyses (TG/DTA; 25–700 °C, 5.0 °C min^−1^, 40 µL platinum crucible, 100 mL min^−1^ dynamic air atmosphere) was carried out on Exstar TG/DTA 6200 thermal analyzer (Seiko Instruments Inc., Chiba, Japan).

Mass spectra were obtained by an LCQ Fleet ion trap mass spectrometer using the positive-ion (ESI+) and negative-ion (ESI–) mode electrospray ionizations (Thermo Scientific; QualBrowser software, version 2.0.7, Thermo Fischer Scientific, Waltham, MA, USA) on the fresh methanol solutions, after 24 h and after 48 h. Hydrolysis studies: the 10 µM (the final concentration) solution of the representative complex **5** in methanol was mixed together with the same volume of water (presence of methanol ensured the solubility of **5**, because carrying out of the experiments in a pure water was prevented by its limited solubility in water). 20 µL of the mixtures was analysed by means of flow injection analysis/mass spectrometry (FIA/ESI-MS) in both the positive and negative ionization modes (fresh methanol solutions, after 24 h and after 48 h).

^1^H-, ^13^C- and ^195^Pt-NMR spectra and two dimensional correlation experiments (^1^H–^1^H gs-COSY, ^1^H–^13^C gs-HMQC, ^1^H–^13^C gs-HMBC, ^1^H–^15^N gs-HMBC; gs = gradient selected, COSY = correlation spectroscopy, HMQC = heteronuclear multiple quantum coherence, HMBC = heteronuclear multiple bond coherence) of the DMF-*d_7_* solutions were recorded at 25 °C on a Varian 400 device (Agilent, Santa Clara, CA, USA) at 400.00 MHz (^1^H), 100.58 MHz (^13^C), 86.00 MHz (^195^Pt) and 40.53 MHz (^15^N). ^1^H and ^13^C spectra were adjusted against the signals of tetramethylsilane (SiMe_4_), ^195^Pt spectra against K_2_[PtCl_6_] in D_2_O (found at 0 ppm) and ^1^H–^15^N gs-HMBC experiments against the residual signals of DMF adjusted to 8.03 ppm (^1^H) and 104.7 ppm (^15^N). The splitting of proton resonances in the reported ^1^H spectra is defined as s = singlet, d = doublet, t = triplet, q = quartet, br = broad band, m = multiplet. Hydrolysis studies: the DMF-*d_7_*/H_2_O (4:1 *v/v*) solution of the representative complex **5** was carried out by means of ^1^H and ^195^Pt NMR after on the fresh solution, and after 24 and 48 h of standing at laboratory temperature.

Theoretical calculations at the DFT and DFT-D3 [20,21] level (B3LYP functional [22], LANLTZ (for Pt [23,24]) and 6-311-G** (for other elements [25]) basis sets) were performed on the representative complex **5**, whose built model (we used the knowledge based on the results of the mentioned analytical techniques together with the known structures of similar complexes) was optimized by the ORCA software package [26].

The human breast adenocarcinoma MCF7 (ATCC HTB-22) and human osteosarcoma HOS (ATCC CRL-1543) cancer cell lines were supplied from European Collection of Cell Cultures. *In vitro* cytotoxicity against the mentioned cell lines was evaluated by an MTT assay [MTT = 3-(4,5‑dimethylthiazol-2-yl)-2,5-diphenyltetrazolium bromide]. The cells were cultured according to the ECACC instructions and they were maintained at 37 °C and 5% CO_2_ in a humidified incubator. The cells were treated with *n*L, **1**–**5** and cisplatin (applied up to 50 μM) for 24 h, using multi-well culture plates of 96 wells. In parallel, the cells were treated with vehicle (DMF; 0.1%, v/v) and Triton X-100 (1%, v/v) to assess the minimal (*i.e.*, positive control) and maximal (*i.e.*, negative control) cell damage, respectively. The MTT assay was measured spectrophotometrically at 540 nm (TECAN, Schoeller Instruments LLC, Praha, Czech Republic). The data were expressed as the percentage of viability, when 100% and 0% represent the treatments with DMF and Triton X-100, respectively. The cytotoxicity data from the cancer cell lines were acquired from three independent experiments (conducted in triplicate) using cells from different passages.

The cellular accumulation experiments (performed in triplicate) of **5** and cisplatin were carried out on MCF7 human cancer cells. The cells were seeded in 100-mm tissue culture dishes (30,000 cells/cm^2^) and incubated overnight (37 °C and 5% CO_2_ in a humidified incubator). After that the cells were treated with **5** (10 μM final concentration), cisplatin (standard; 10 μM final concentration) or with the pure medium (control) for 24 h. The cells were washed with PBS (2 × 2 mL), harvested by trypsin treatment, collected and centrifuged in PBS. The supernatants were discarded after centrifugation and pellets were digested in hydrochloric acid using a microwave system (Monowave300, Anton Paar, Praha, Czech Republic). The platinum content was determined by ICP-MS (ICP-MS spectrometer 7700x, Agilent). The obtained values were corrected for adsorption effects.

### 3.2. Synthesis

K_2_[Pt(ox)_2_]∙2H_2_O (0.25 mmol) dissolved in a minimum volume of hot distilled water was added to 0.5 mmol of the appropriate *N*6-benzyladenosine derivative (*n*L) dissolved in a minimum volume of hot methanol. The reaction mixture was stirred at 60 °C for five days and another two days at laboratory temperature. Slow evaporation of methanol led to the formation of white to light yellow products, which were filtered off and washed by distilled water (5 mL) and methanol (5 mL). The obtained complexes **1**–**5** were dried at the temperature of 40 °C and stored in desiccator over silica gel.

The recrystallization of the products from different organic solvents (DMF, acetone, chloroform *etc.*) in combination with different techniques (cooling of hot solutions, slow evaporation, diffusion method) did not lead to the formation of single-crystals suitable for single-crystal X-ray analysis.

*Bis{N6-(2-methoxybenzyl)adenosine)-κN7}(oxalato-κ^2^O,O')platinum(II) sesquihydrate* (**1**). C_38_H_42_N_10_O_14_Pt∙1.5H_2_O; white solid; yield 75%; ^1^H-NMR (DMF-d_7_): *δ* = 9.24 (s, CH-8), 8.90 (t, *J* = 6.3, NH-6), 8.35 (s, CH-2), 7.24 (t, *J* = 7.4, CH-13), 7.23 (d, *J* = 7.3, CH-15), 7.03 (d, *J* = 8.9, CH-12), 6.79 (t, *J* = 7.7, CH-14), 6.12 (d, *J* = 4.9, CH-16), 5.82 (br, OH-17'), 5.46 (br, OH-20'), 5.38 (br, OH-18'), 4.92 (d, *J* = 6.1, CH_2_-9), 4.78 (t, *J* = 4.9, CH-17), 4.38 (t, *J* = 4.6, CH-18), 4.15 (q, *J* = 3.6, CH-19), 3.92 (s, CH_3_-11'), 3.85 (m, CH-20a), 3.74 (m, CH-20b) ppm; ^13^C-NMR (DMF-d_7_): *δ* = 165.59 (C-ox), 157.17 (C-11), 153.90 (C-6), 152.98 (CH-2), 147.98 (C-4), 143.62 (CH-8), 128.09 (CH-13), 127.26 (CH-15), 126.54 (C-10), 120.41 (CH-14), 117.04 (C-5), 110.33 (CH-12), 90.37 (CH-16), 86.92 (CH-19), 74.70 (CH-17), 70.71 (CH-18), 61.68 (CH_2_-20), 55.30 (CH_3_-11'), 39.64 (CH_2_-9) ppm; ^15^N NMR (DMF-d_7_): *δ* = 90.64 (NH-6), 127.63 (N-7), 178.16 (N-9), 223.88 (N-3), 235.84 (N-1) ppm; ^195^Pt NMR (DMF-d_7_): *δ* = −1691.25 ppm; ESI MS (30 eV): *m/z* = 1096.6 (M + K), 1080.2 (M + Na), 1058.3 (M + H), 1056.2 (M − H), 388.3 (*2OMe*L + H); Anal. Calc.: C, 42.1; H, 4.2; N, 12.9. Found: C, 42.0; H, 4.0; N, 12.9%.

*Bis{N6-(4-methoxybenzyl)adenosine)-κN7}(oxalato-κ^2^O,O')platinum(II) sesquihydrate* (**2**). C_38_H_42_N_10_O_14_Pt∙1.5H_2_O; white solid; yield 70%; ^1^H-NMR (DMF-d_7_): *δ* = 9.19 (s, CH-8), 8.93 (t, *J* = 5.9, NH-6), 8.35 (s, CH-2), 7.32 (d, *J* = 8.6, CH-12,14), 6.82 (d, *J* = 8.6, CH-11,15), 6.09 (d, *J* = 5.1, CH-16), 5.80 (br, OH-17'), 5.37 (br, OH-18'), 4.84 (d, *J* = 5.9, CH_2_-9), 4.74 (t, *J* = 4.7, CH-17), 4.35 (t, *J* = 4.3, CH-18), 4.13 (q, *J* = 3.5, CH-19), 3.86 (m, CH-20a), 3.83 (m, CH-20b), 3.77 (s, CH_3_-13') ppm; ^13^C-NMR (DMF-d_7_): *δ* = 165.53 (C-ox), 158.70 (C-13), 153.76 (C-6), 152.66 (CH-2), 147.88 (C-4), 143.47 (CH-8), 131.01 (C-10), 128.53 (CH-12,14), 116.89 (C-5), 113.70 (CH-11,15), 90.23 (CH-16), 86.79 (CH-19), 74.66 (CH-17), 70.58 (CH-18), 61.56 (CH_2_-20), 54.87 (CH_3_-11´), 43.55 (CH_2_-9) ppm; ^15^N-NMR (DMF-d_7_): *δ* = 95.48 (NH-6), 127.80 (N-7), 178.15 (N-9), 223.74 (N-3), 236.18 (N-1) ppm; ESI MS (30 eV): *m/z* = 1096.2 (M + K), 1080.2 (M + Na), 1056.1 (M − H), 388.1 (*4OMe*L + H); Anal. Calc.: C, 42.1; H, 4.2; N, 12.9. Found: C, 42.0; H, 4.0; N, 12.9%.

*Bis{N6-(4-methylbenzyl)adenosine)-κN7}(oxalato-κ^2^O,O')platinum(II) sesquihydrate* (**3**). C_38_H_42_N_10_O_12_Pt∙1.5H_2_O; white solid; yield 80%; ^1^H-NMR (DMF-d_7_): *δ* = 9.22 (s, CH-8), 8.98 (t, *J* = 6.3, NH-6), 8.35 (s, CH-2), 7.27 (d, *J* = 8.3, CH-11,15), 7.07 (d, *J* = 7.4, CH-12,14), 6.10 (d, *J* = 5.1, CH-16), 5.60 (br, OH-17'), 5.40 (br, OH-20'), 5.30 (br, OH-18'), 4.87 (d, *J* = 6.2, CH_2_-9), 4.76 (t, *J* = 4.8, CH-17), 4.37 (t, *J* = 4.3, CH-18), 4.14 (q, *J* = 3.2, CH-19), 3.86 (m, CH-20a), 3.75 (m, CH-20b), 2.28 (s, CH_3_-13') ppm; ^13^C-NMR (DMF-d_7_): *δ* = 165.68 (C-ox), 153.88 (C-6), 152.86 (CH-2), 148.03 (C-4), 143.62 (CH-8), 136.26 (C-10), 136.18 (C-13), 129.08 (CH-12,14), 127.24 (CH-11,15), 117.04 (C-5), 90.39 (CH-16), 86.95 (CH-19), 74.77 (CH-17), 70.76 (CH-18), 61.72 (CH_2_-20), 43.95 (CH_2_-9), 20.44 (CH_3_-13') ppm; ^15^N-NMR (DMF-d_7_): *δ* = 94.91 (NH-6), 128.00 (N-7), 178.08 (N-9), 223.84 (N-3), 236.40 (N-1) ppm; ^195^Pt-NMR (DMF-d_7_): *δ* = −1688.92 ppm; ESI MS (30 eV): *m/z* = 1064.1 (M + K), 1048.2 (M + Na), 1024.3 (M − H), 372.1 (*4Me*L + H), 370.2 (*4Me*L − H); Anal. Calc.: C, 43.4; H, 4.3; N, 13.3. Found: C, 43.3; H, 4.0; N, 13.4%. 

*Bis{N6-(2-chlorobenzyl)adenosine)-κN7}(oxalato-κ^2^O,O')platinum(II) sesquihydrate* (**4**). C_36_H_36_N_10_Cl_2_O_12_Pt∙1.5H_2_O; white solid; yield 80%; ^1^H-NMR (DMF-d_7_): *δ* = 9.36 (s, CH-8), 9.11 (t, *J* = 6.9, NH-6), 8.35 (s, CH-2), 8.48 (d, *J* = 7.6, CH-15), 8.38 (d, *J* = 8.0, CH-12), 7.29 (t, *J* = 7.6, CH-14), 7.15 (t, *J* = 7.6, CH-13), 6.17 (d, *J* = 4.9, CH-16), 5.87 (d, *J* = 5.2, OH-17'), 5.49 (t, *J* = 5.6, OH-20'), 5.40 (d, *J* = 4.7, OH-18'), 5.00 (d, *J* = 6.2, CH_2_-9), 4.80 (q, *J* = 4.9, CH-17), 4.42 (q, *J* = 4.4, CH-18), 4.18 (q, *J* = 3.7, CH-19), 3.90 (m, CH-20a), 3.79 (m, CH-20b) ppm; ^13^C-NMR (DMF-d_7_): *δ* = 165.81 (C-ox), 153.84 (CH-2), 152.82 (C-6), 148.13 (C-4), 143.63 (CH-8), 136.28 (C-10), 131.93 (C-11), 129.15 (CH-15), 128.39 (CH-12), 127.84 (CH-13), 127.32 (CH-14), 117.11 (C-5), 90.26 (CH-16), 86.82 (CH-19), 74.77 (CH-17), 70.61 (CH-18), 61.60 (CH_2_-20), 41.97 (CH_2_-9) ppm; ^15^N-NMR (DMF-d_7_): *δ* = 88.32 (NH-6), 127.63 (N-7), 178.18 (N-9), 224.86 (N-3), 235.91 (N-1) ppm; ^195^Pt-NMR (8DMF-d_7_): *δ* = −1697.63 ppm; ESI MS (30 eV): *m/z* = 1104.3 (M + K), 1088.3 (M + Na), 1064.1 (M − H), 392.2 (*2Cl*L + H), 390.3 (*2Cl*L − H); Anal. Calc.: C, 39.5; H, 3.6; N, 12.8. Found: C, 39.2; H, 3.1; N, 12.6%.

*Bis{N6-(4-fluorobenzyl)adenosine)-κN7}(oxalato-κ^2^O,O')platinum(II) sesquihydrate* (**5**). C_36_H_36_N_10_F_2_O_12_Pt∙1.5H_2_O; white solid; yield 65%; ^1^H-NMR (DMF-d_7_): *δ* = 9.24 (s, CH-8), 9.06 (t, *J* = 6.3, NH-6), 8.36 (s, CH-2), 7.49 (m, CH-11,15), 7.11 (m, CH-12,14), 6.12 (d, *J* = 5.2, CH-16), 5.87 (br, OH-17'), 5.47 (br, OH-20'), 5.14 (br, OH-18'), 4.93 (d, *J* = 6.3, CH_2_-9), 4.76 (t, *J* = 4.9, CH-17), 4.38 (sxt, *J* = 4.6, CH-18), 4.15 (sxt, *J* = 3.2, CH-19), 3.84 (m, CH-20a), 3.75 (m, CH-20b) ppm; ^13^C-NMR (DMF-d_7_): *δ* = 165.76 (C-ox), 163.02, 160.61 (C-13), 153.81 (CH-2), 152.48 (C-6), 148.00 (C-4), 143.61 (CH-8), 135.45 (C-10), 129.65, 129.57, 129.24, 129.16 (CH-11,15), 116.97 (C-5), 115.11, 115.08, 114.90, 114.87 (CH-12,14), 90.28 (CH-16), 86.84 (CH-19), 74.75 (CH-17), 70.62 (CH-18), 61.60 (CH_2_-20), 43.69 (CH_2_-9) ppm; ^15^N-NMR (DMF-d_7_): *δ* = 93.84 (NH-6), 127.52 (N-7), 178.12 (N-9), 224.03 (N-3), 235.88 (N-1) ppm; ^195^Pt-NMR (DMF-d_7_): *δ* = −1688.68 ppm; ESI MS (30 eV): *m/z* = 1071.9 (M + K), 1056.2 (M + Na), 1032.2 (M − H), 376.3 (*4F*L + H), 374.3 (*4F*L − H); Anal. Calc.: C, 40.8; H, 3.7; N, 13.2. Found: C, 40.3; H, 3.4; N, 13.1%.

The IR and Raman spectroscopy results can be found in the Supplementary Materials.

## 4. Conclusions

A series of five platinum(II) oxalato complexes **1**–**5** with the general formulae [Pt(ox)(*n*L)_2_]·1.5H_2_O was synthesized and characterized by relevant methods. The absence of single-crystals suitable for a crystallographic study was compensated by a detailed NMR study involving the multinuclear (^1^H, ^13^C, ^15^N, ^195^Pt) and two-dimensional NMR spectroscopy, which clearly proved the monodentate N7-coordination mode (*Δδ* = *ca* −113 ppm for N7 in ^15^N-NMR spectra) of *N*6-benzyl-adenosine-based ligands and bidentate coordination of the oxalate dianion. One of the main goals of the work was to find whether the platination of *N*6-benzyladenosine derivatives affect *in vitro* cytotoxicity of the final [Pt(ox)(*n*L)_2_]·1.5H_2_O complexes **1**–**5** against HOS osteosarcoma and MCF7 breast adenocarcinoma human cancer cell lines. The results of the testing revealed that although usually very sensitive cell lines to the action of platinum complexes have been used, all the complexes have been found as inactive up to the concentrations of 50.0 µM (for compounds **1** and **2**) and 20.0 µM (for compounds **3**, **4** and **5**), given by their limited solubility. The *in vitro* cytotoxic inactivity of the studied complexes corresponds with low cellular accumulation (*ca* 50-fold lower than cisplatin) as observed on the representative complex **5**, which was moreover found (ESI-MS, NMR) as resistant to the hydrolytic processes.

## Abbreviations

*Δδ*: coordination shift (calculated as *δ_complex_* − *δ_ligand_*)
COSY: correlation spectroscopy
MTT: 3-(4,5-dimethylthiazol-2-yl)-2,5-diphenyltetrazolium bromide
gs: gradient selected
HMBC: heteronuclear multiple bond coherence
HMQC: heteronuclear multiple quantum coherence
MCF7: human breast adenocarcinoma cancer cell line
HOS: human osteosarcoma cancer cell line
*2Cl*L: *N*6-(2-chlorobenzyl)adenosine
*2OMe*L: *N*6-(2-methoxybenzyl)adenosine
*4F*L: *N*6-(4-fluorobenzyl)adenosine
*4OMe*L: *N*6-(4-methoxy- benzyl)adenosine
*4Me*L: *N*6-(4-methylbenzyl)adenosine
*n*L: *N*6-(substituted-benzyl)-adenosine derivatives in general
ESI–: negative-ion mode electrospray ionization
ESI+: positive-ion mode electrospray ionization

## Supplementary Materials

Supplementary materials can be accessed at: http://www.mdpi.com/1420-3049/19/3/3832/s1.
