# Supplementary material for: Platinum(II) Oxalato Complexes Involving Adenosine-Based N-Donor Ligands: Synthesis, Characterization and Cytotoxicity Evaluation
Source: Molecules. 2014 Mar 24;19(3):3832–47. doi: 10.3390/molecules19033832 (PMC6271696; doi:10.3390/molecules19033832)
Supplement: Supplementary file 1 [file molecules-19-03832-s001.pdf]

Supplementary Materials

Figure S1. The results of simultaneous TG/DTA thermal analyses of **1** and **3**, as representatives, given with the calculated and found weight losses for the dehydration (step 1 in green) and thermal decomposition of the dehydrated complexes (step 2 in red) to the PtO as the final product.

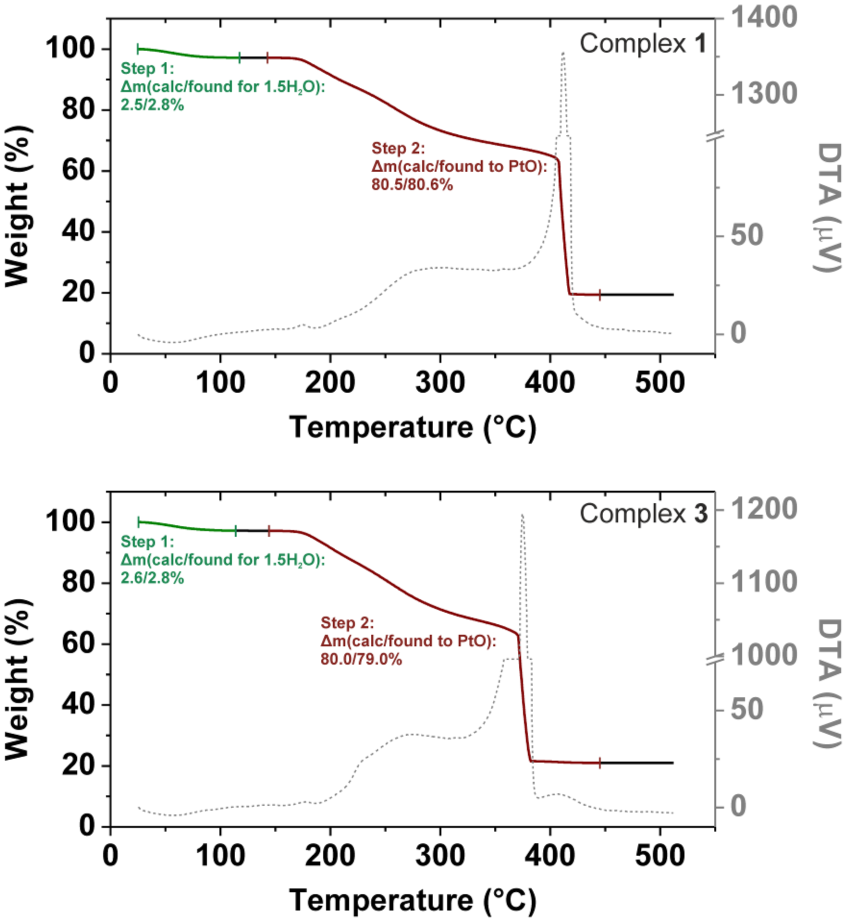

Figure S2. The IR spectrum of the complex **4** and Raman spectrum of the complex **1**.

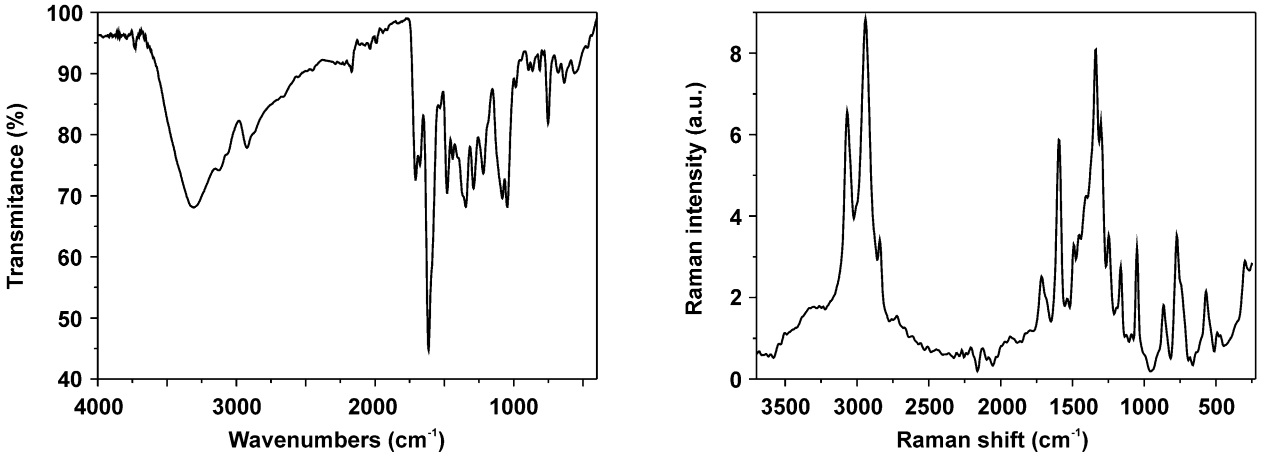

Figure S3. The results of the ^{1}H-NMR (**A**), ^{13}C-NMR (**B**), APT (**C**) and ^{195}Pt NMR (**D**) spectroscopy of the representative complex **1**.

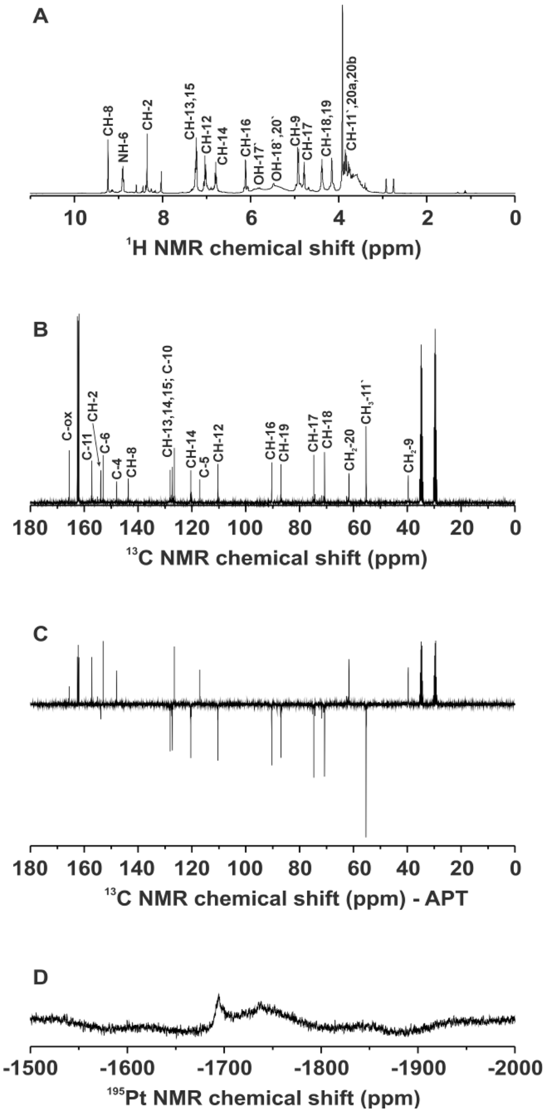

Figure S4. The results of the ^{1}H–^{13}C gs-HMBC (**A**) and ^{1}H–^{13}C gs-HMQC (**B**) experiments of the representative complex **1**.

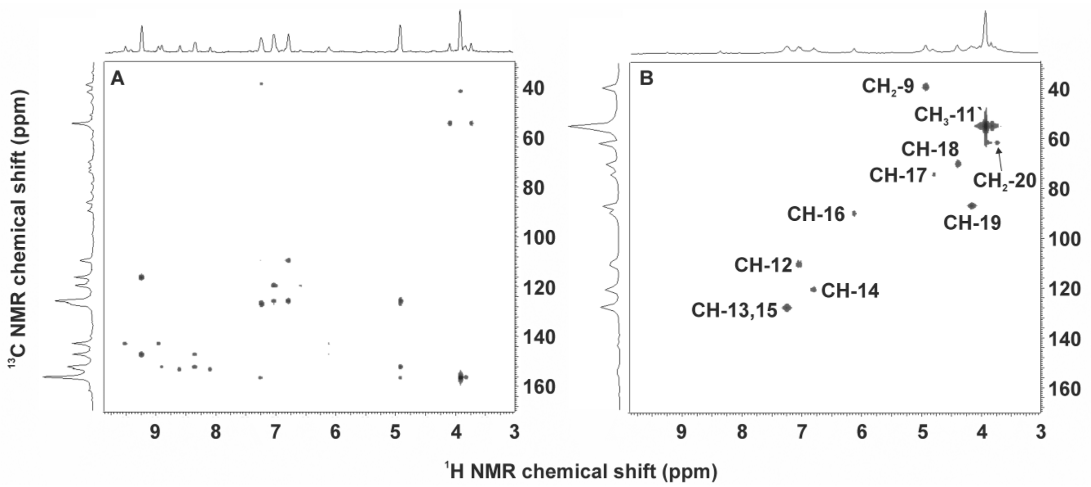

Figure S5. Parts of the ESI+ mass spectra of **5** in methanol (**A**) and in methanol/water mixture (1:1 *v/v*) as recorded on fresh solutions (**B**), after 24 h (**C**) and after 48 h (**D**), which contain the peaks of the $\{[Pt(ox)(4FL)_2]+H\}^+$ and $\{[Pt(ox)(4FL)_2]+Na\}^+$ at 1,034.2 *m/z*, and 1,056.2 *m/z*, respectively.

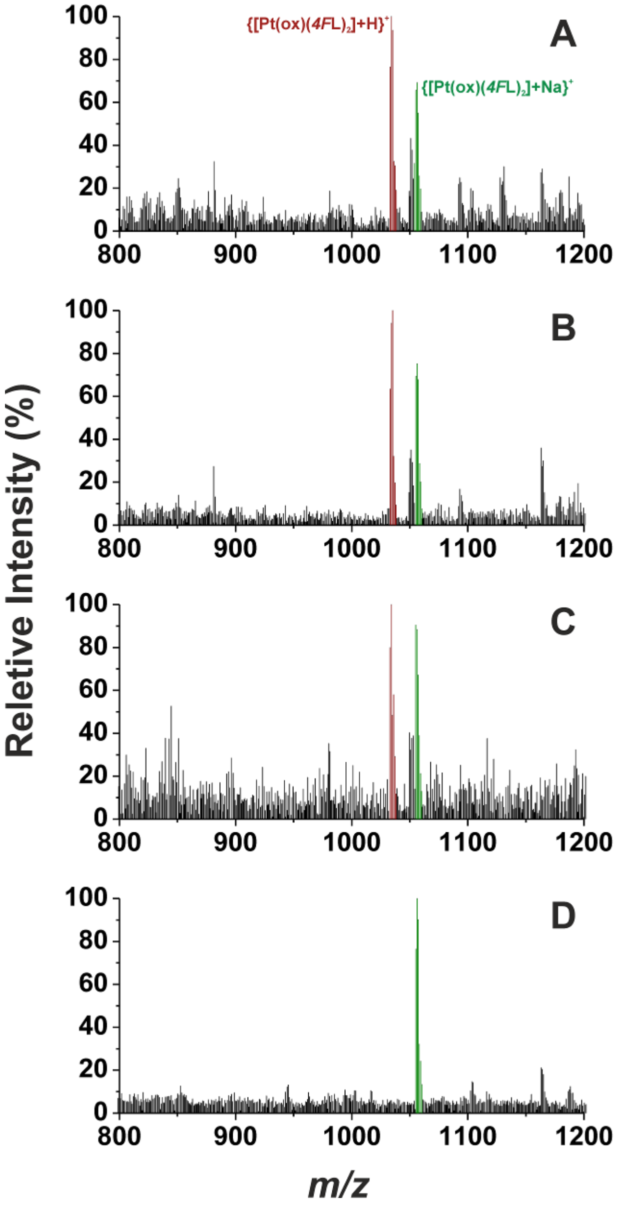

Figure S6. The results of the ^{1}H-NMR experiments of the representative complex **1** as obtained on its DMF-d_7 (**A**) and DMF-d_7/H_2O solutions recorded on fresh solution (**B**), after 24 h (**C**) and after 48 h (**D**).

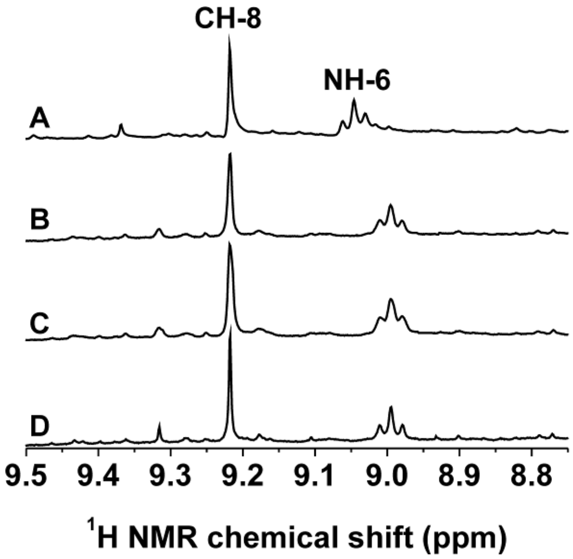

The results of IR and Raman spectroscopy of the complexes 1–5

1: IR (ATR): 223s, 279s, 399s, 445vs, 458vs, 487s, 495s, 505s, 526vs, 538s, 558m cm^{-1}; IR (ATR): 463w, 488w, 526w, 567w, 630w, 675w, 709w, 752m, 785w, 811m, 863m, 894m, 984m, 1024s, 1049s, 1078s, 1118s, 1239s, 1290s, 1348s, 1366s, 1407m, 1437m, 1469s, 1487s, 1534m, 1586vs, 1613vs, 1692m, 1710s, 2838s, 2929s, 3116s, 3326vs cm^{-1}; Raman: 299m, 469w, 492w, 571m, 746m, 772m, 865w, 1051m, 1163m, 1246m, 1298s, 1338vs, 1405s, 1456m, 1491m, 1535w, 1591s, 1685w, 1714m, 2840m, 2940vs, 3069vs cm^{-1}.

2: IR (ATR): 467w, 517w, 568w, 635w, 675w, 713w, 755w, 789m, 811m, 895m, 985m, 1028s, 1056s, 1079s, 1176s, 1239vs, 1294s, 1350s, 1371s, 1464s, 1485s, 1511vs, 1586vs, 1610vs, 1672s, 1707s, 2837s, 2930s, 3120s, 3319vs cm^{-1}; Raman: 444m, 568m, 638m, 743m, 821m, 848m, 1178m, 1253m, 1313s, 1337vs, 1360s, 1460s, 1586s, 1613s, 1707m, 2841s, 2934vs, 3009s, 3061vs cm^{-1}.

3: IR (ATR): 279s, 354m, 377m, 395s, 454vs, 470vs, 506s, 524s, 535s, 572s cm^{-1}; IR (ATR): 470w, 526w, 548w, 567w, 632w, 747w, 789m, 808w, 863m, 894m, 984m, 1054s, 1078s, 1218s, 1291s, 1344s, 1371s, 1409s, 1483s, 1515m, 1584vs, 1611vs, 1669m, 1710s, 2866s, 2921s, 3123s, 3304vs cm^{-1}; Raman: 308m, 363m, 567m, 642w, 739m, 812w, 838m, 1184w, 1204m, 1300s, 1337vs, 1371s, 1457m, 1486m, 1542m, 1587m, 1614s, 1674w, 1702m, 2926vs, 3054s cm^{-1}.

4: IR (ATR): 382m, 415s, 435s, 454s, 465s, 494s, 508s, 534vs, 562s cm^{-1}; IR (ATR): 463w, 567w, 635w, 679w, 751m, 790w, 811w, 865w, 894w, 984w, 1049s, 1081s, 1182m, 1219m, 1291s, 1345s, 1369s, 1442,. 1483s, 1535m, 1614vs, 1679m, 1708s, 2928m, 3129m, 3063m, 3305s cm^{-1}; Raman: 558w, 744m, 871w, 1041m, 1161m, 1206m, 1298s, 1338vs, 1489s, 1590s, 1697m, 2936vs, 3065vs cm^{-1}.

5: IR (ATR): 389m, 418s, 433s, 458s, 482vs, 520s, 533s, 543m, 559m cm^{-1}; IR (ATR): 483w, 567m, 635m, 711w, 762m, 789m, 825m, 895m, 984m, 1017m, 1055s, 1079s, 1157s, 1217vs, 1291s, 1344s, 1371s, 1485s, 1508, 1614vs, 1680s, 1707s, 2926s, 3116s, 3305vs cm^{-1}; Raman: 311m, 447w, 563m, 636m, 746m, 852s, 1160m, 1219s, 1339vs, 1487s, 1532m, 1587vs, 1680m, 1713m, 2945vs, 3070vs cm^{-1}.
